# Experience-Dependent Effects to Situational Awareness in Police Officers: An Eye Tracking Study

**DOI:** 10.3390/ijerph19095047

**Published:** 2022-04-21

**Authors:** Juha-Matti Huhta, Paula M. Di Nota, Veikko Surakka, Poika Isokoski, Eero Ropo

**Affiliations:** 1 Police University College of Finland, 33721 Tampere, Finland; 2 Faculty of Education and Culture, Tampere University, 33720 Tampere, Finland; eero.ropo@tuni.fi; 3 Department of Psychology, University of Toronto Mississauga, Mississauga, ON L5L 1C6, Canada; paula.dinota@utoronto.ca; 4 Faculty of Information Technology and Communication Sciences, Tampere University, 33720 Tampere, Finland; veikko.surakka@tuni.fi (V.S.); poika.isokoski@tuni.fi (P.I.)

**Keywords:** situational awareness, police, eye tracking, fixation, expertise, police encounters, visuomotor neuroscience

## Abstract

Police work requires making suitable observations which form the basis of situational awareness (SA) of the encounter in progress. Incomplete early-stage SA (i.e., perception) can lead to errors in subsequent judgement and decision-making that can have severe consequences for performance, learning, and occupational health. SA in police contexts is still relatively understudied and requires closer examination using objective measures. The current preliminary study aimed to measure the gaze and fixation patterns among novice and expert police officers to understand early-stage SA at different levels of professional experience. Participants included 23 novices (10 early, 13 intermediate) and 11 experienced officers and instructors in tactics and use of force. Visit duration and fixation order were measured while participants viewed various static images of staged encounters. Results showed that all participants fixated longer on targets compared to the periphery, and fixated earlier on suspects’ faces compared to hands, bodies, or the environment. Further, experts fixated earlier on hands and spent less time scanning the environment than early novices. The current findings reveal eye movement patterns while officers engaged in typical police encounters. Future research can inform evidence-based police training to achieve optimal SA and minimize negative outcomes in training and operational field settings.

## 1. Introduction

Police work consists of evaluating and resolving complex situations, quite often under time pressure and uncertainty. Potentially violent encounters are especially demanding and require rapid assessment and effective judgement, decision-making, and actions [1,2,3]. Therefore, where and how officers visually scan the unique environments they are faced with is a critical professional skill. Repeated and prolonged exposure to potentially traumatic events place police officers at greater risk for developing operational stress injuries (OSIs), which include elevated symptoms of depression, anxiety, and post-traumatic stress disorder (PTSD) [4,5], and exhibiting suicidal thoughts and behaviors [6]. Potentially traumatic exposures may include negative outcomes in operational field settings and/or training contexts, which can diminish officers’ confidence and compromise subsequent performance. Therefore, developing effective strategies for navigating critical incidents is an important goal for police work and education.

Situational awareness (SA) (sometimes also referred to as ‘situation awareness’) is perhaps the most important skill required by police and other first responders, and forms the basis of judgement, decision-making, and action [7]. Although it has been extensively studied in several industries (e.g., sports, aviation, military command, medicine) at the individual and group level ([8,9], for review, see [10]), there are very few empirical studies that examine, conceptualize, or define SA in policing contexts. One of the main definitions of SA currently employed by police instructors is based on the work of Endsley [11], who divides SA into three components: perception, comprehension, and projection. The current study will focus on early-stage SA, as the information which officers are able to gather via perception will inform downstream comprehension, projection, and decision-making.

Of the very few previous studies that measure SA in police, operational definitions and details on measurement criteria are lacking [12]. In both research and practice, SA is commonly scored on a subjective scale, which makes instructor evaluations vulnerable to potential biases [13]. In light of limited SA police research, we look to studies that measure objective eye movement patterns to understand how training and experience can shape early-stage SA. By understanding how expert police gather essential information, evidence-based SA training can be developed.

Expert police officers (i.e., more field experience and specialized training [7]) and trained emergency responders have shown different patterns of directing their gaze compared to untrained individuals and novice police. Based on a systematic review of eye tracking studies involving police and law enforcement, Heusler and Sutter [14] state that visual perception is strongly related to expertise, such that experts are better able to attend to and focus on essential information (see also [15]). Furthermore, experts’ domain-specific knowledge and experience facilitate improved early SA and decision-making under stressful conditions. Specifically, Vickers and Lewinski [2] found that expert officers shot more accurately and made fewer decision-making errors than beginners during live “shoot/no-shoot” scenarios. Using eye tracking equipment, this study also showed that the experts were more likely to look at locations where the firearm could have been hidden in the suspect′s clothes, while the novices′ eyes were directed to a wider area. Recently, Heusler and Sutter [16] compared gaze and fixation behaviors between tactical unit and patrol officers while they performed video-based “shoot/no-shoot” scenarios. Expert tactical officers spent significantly shorter time fixating on targets’ faces, and longer on the hands and hip areas than patrol officers. However, every scenario included in this study involved a weapon, which may have primed participants to search for this highly salient target in every situation.

When comparing visuomotor behaviors during high versus low threat simulations, police officers demonstrate increased blinking and diverting their gaze, and head movements away from target locations under high threat [17]. Following stressful real-world encounters (i.e., officer-involved shootings), officers report several perceptual distortions including tunnel vision and slowed sense of time [18]. High threat can also impact the later stages of SA—comprehension and projection—including a phenomenon referred to as ‘affective realism’, whereby objects such as a radio or cellphone are mistakenly perceived as a firearm [12,19]. However, training under stressful conditions has been shown to improve deficits in visuomotor gaze behavior, SA, and lethal force decision-making [12,20,21]. In police use of force and tactics training, SA may not be consistently trained as a finite skill despite its relevance to threat assessment, decision-making, and motor skills development [7,22]. Therefore, the development of SA should focus on effective methods of exploration to identify essential features of the situation as it continues to unfold [23].

The purpose of the current study was to understand expert models of early-stage SA (i.e., perception) in police, and specifically, how they search for critical information in various typical police encounters. To clarify, this study was not intended to evaluate SA during high-stress situations where officers needed to make split-second shoot/no-shoot decisions, nor where their gaze behaviors may be impacted by stress physiology. Rather, the current study sought to measure early-stage SA while viewing a variety of encounters that police officers face daily, including critical ones. Early-stage SA was operationalized by objective gaze and fixation behaviors during a scene perception task. By comparing expert visuomotor patterns to those among novice police trainees, the current findings can provide evidence-based insights for police training of SA and tactics.

## 2. Materials and Methods

### 2.1. Participants

A total of 34 participants took part in the study, and were divided into expert (*n* = 11, all male) and novice (*n* = 23, 11 female) groups based on their experience (see Table 1). Novices were further subdivided based on their studies carried out, such that students in periods 1 to 4 were assigned to the Novice 1 (N1) group, and students in periods 5 to 6 (i.e., had all or almost all tactical training and were just about to start their practical training in the field) were assigned to the Novice 2 (N2) group [24]. Novice students were invited to participate in the study through an email distributed in the institute’s internal email system (Wilma). The research recruitment message sent to the students indicated that eligibility required that the participant does not have previous training or work experience in the security sector. Expert police officers had an average of 16.7 years of experience as police officers (*SD* = 3.9, range: 12−25), and an average of 8.0 years of experience in special units (*SD* = 2.2, range: 3.5−10) including K-9, special response units, and instructors in tactics and use of force. Experts were invited to participate in the study based on their geographical location and availability.

The study was approved by the research ethics board of the Police University College of Finland.

### 2.2. Procedure

**Figure 1:**
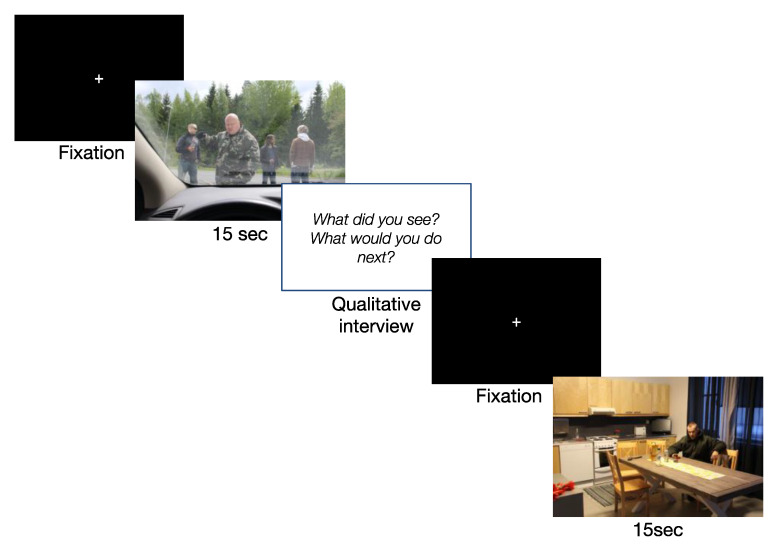
Experimental procedure. Note that interview questions following each image were the same and were not presented on the computer screen. Qualitative analyses of interview data will be presented in a separate study.

The study was conducted at the Police University College of Finland in February 2018 using a computer connected to a 17-inch monitor and built-in eye tracker system (Tobii T60, software version Tobii Pro Lab 1.64). Following informed consent, fitting, and calibration of the eye tracker, participants were told that they will be presented with 13 static photographs of staged encounters of a confrontational nature (see Supplementary Materials). As visualized in Figure 1, participants were given 15 s to observe each image during which time their gaze activity was recorded (see Measures). Following presentation of each image, participants were asked specific questions to probe their SA and tactical decision-making. The qualitative results of the interview data will be presented in a separate study. Once participants had finished answering the interview questions, they were asked to fixate on the center of the computer screen and a button was pressed to present the next image.

### 2.3. Measures

Using the Gazeplot feature in the Tobii Pro Lab software system, an expert police instructor (J-MH) identified boundary regions around several areas of interest (AOIs) for each image, including the target person(s) and the peripheral environment. Within the target AOIs, further subareas of interest (subAOIs) were marked around suspect faces, hands, and body (i.e., excluding the face and hands), which are most tactically relevant to police SA in critical incidents. Data were obtained within 5 s from stimulus onset for each image to capture early SA (i.e., initial visual perception).

**Figure 2:**
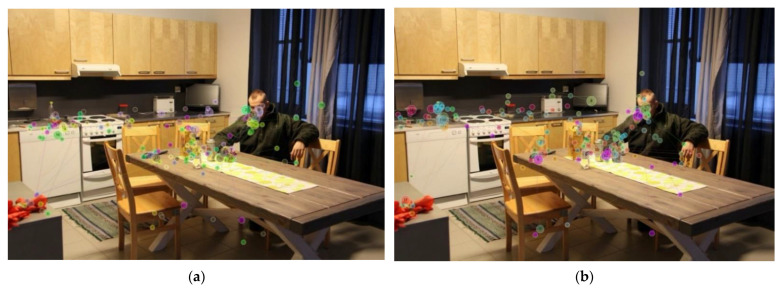
Sample eye tracking data. In both images, each individual circle represents a participant’s point of fixation. The size of each circle represents visit duration, with larger circles representing longer durations. The number within each circle represents the order of fixation following stimulus onset. The color of each circle corresponds to a specific participant: (**a**) Visualized eye tracking data for Novice 1 participants; (**b**) Visualized eye tracking data for expert participants.

Situational awareness was operationalized by the following objective measures (Figure 2):

(1) *Visit duration*: The total time spent (in seconds) fixating or moving the eyes searching within overall target and environment AOIs.

(2) *Fixation order*: The first fixation point within a predefined AOI was marked and assigned a weighted value of 100. All subsequent fixation points received half of the previous value, such that the second fixation point received a value of 50, the third fixation value was 25, the fourth fixation value was 12.5, and the fifth fixation value was 6.25. Only 2 instances recorded a seventh fixation valued at 3.125 (0.3% of 638 cases). The initial fixation at the center of the image at its onset was excluded, and no target AOIs were located at central fixation. If participants did not fixate within a predefined AOI within the first 5 s, it received a value of zero. Descending weights were assigned for later fixations to reflect decreasing salience in early SA and scene perception among police. Data were averaged across individual images to compute mean weights for face, hand (left and/or right), body, and environment subAOIs.

Due to limitations in the eye tracking equipment and stimulus design (see Limitations in Discussion), visit duration data was obtained from 7 of 13 images, and fixation order data was obtained from 4 of 13 images. For example, Figures S1 and S2 were excluded because target subAOIs were too small, numerous, and close together, and the eye tracker was not sensitive enough to distinguish between them to register valid fixation duration or order values.

### 2.4. Statistical Analyses

Visit duration and fixation order data were entered into SPSS (Version 25, IBM Corp., Armonk, NY, USA) for statistical analyses. Continuous visit duration data were normally distributed according to Shapiro–Wilk tests (*ps* > 0.05). Therefore, a two-way repeated measures ANOVA was used to examine the main effects and interaction between AOIs (target, environment) and group (Novice 1, Novice 2, Expert). Visit duration data for each image was also descriptively analyzed and presented. Ordinal fixation order data were compared using non-parametric tests (i.e., Kruskal–Wallis, pairwise comparisons using Mann–Whitney U test) to evaluate between-group differences among experts and both novice groups. Within-group differences in fixation order between face, hands, body, and environment were tested using pairwise Wilcoxon Signed-Rank tests separately for each group. Due to the highly novel and exploratory nature of the current dataset, significance criteria were set at *p* < 0.05 with Bonferroni-corrected values reported where available.

## 3. Results

### 3.1. Visit Duration

**Figure 3:**
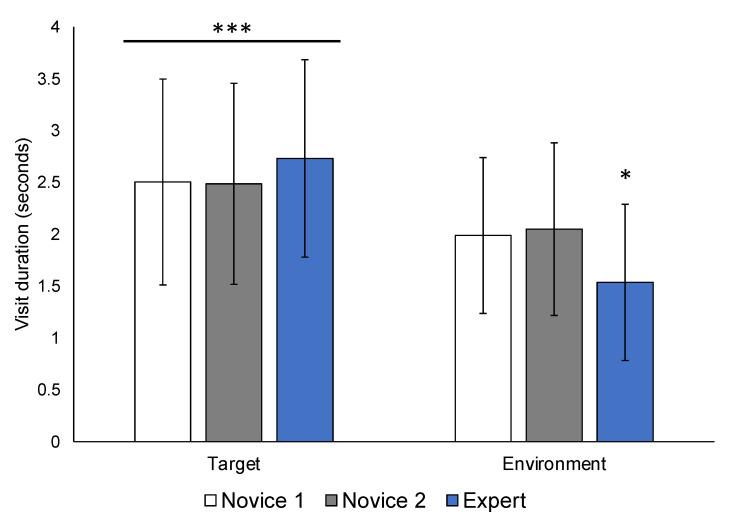
Average visit duration for target versus environment areas of interest. The mean time (in seconds) spent fixating on or scanning pre-defined target (left bars) and environment areas of interest (right bars) for 7 images are presented for each group (shown in different colours). All groups spent significantly more time fixating and scanning target areas compared to the environment. Experts spent significantly less time viewing the environment compared to both novice groups. *** *p* < 0.001, * *p* < 0.05.

A significant main effect of AOI revealed that all participants spent significantly more time scanning and fixating target areas compared to the environment with a large effect size (F(1,31) = 30.034, *p* < 0.001, η^2^ = 0.492) (Figure 3). The main effect of group was not significant (F(2,31) = 1.384, *p* = 0.266, η^2^ = 0.082). However, a significant interaction between AOI and group (F(2,31) = 3.339, *p* = 0.049, η^2^ = 0.177) revealed that experts spent significantly less time fixating the environment compared to Novice 1 (*p_Bonf_* = 0.050) and Novice 2 (*p_Bonf_* = 0.015), with no differences in visit duration for target AOIs.

**Figure 4:**
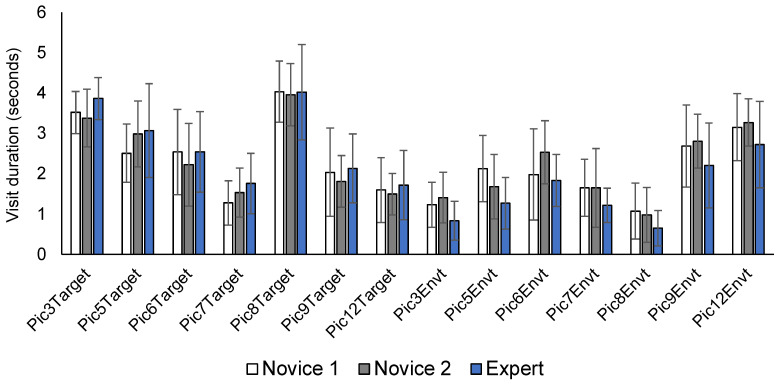
Visit duration for target versus environment areas of interest for seven individual images (see Supplementary Materials).

Within-subject analyses comparing visit duration in target versus environment AOIs were not conducted, as differences in these variables depend on the specific context of each image (Figure 4). For example, all groups spent more time looking at the target person in Pictures 3 and 8 (see Figures S3 and S4 in Supplementary Materials), while the environment was attended to more in Pictures 9 (Figure S5) and 12 (see Figure 2 above). Differences in target versus environment fixation that are consistent across all participants can be accounted for by situational differences that are unique to each image. For example, Pictures 3 and 8 (Figures S3 and S4) mainly feature a central target person with relatively few peripheral cues. In contrast, Pictures 9 and 12 feature relatively more visual cues in the environment, which directed all participants to spend more time visually exploring the surroundings. As we were not interested in evaluating the differences in gaze patterns *between images*, visit duration data were averaged across individual images to ascertain differences in gaze and fixation patterns between novice and expert police.

### 3.2. Fixation Order

**Figure 5:**
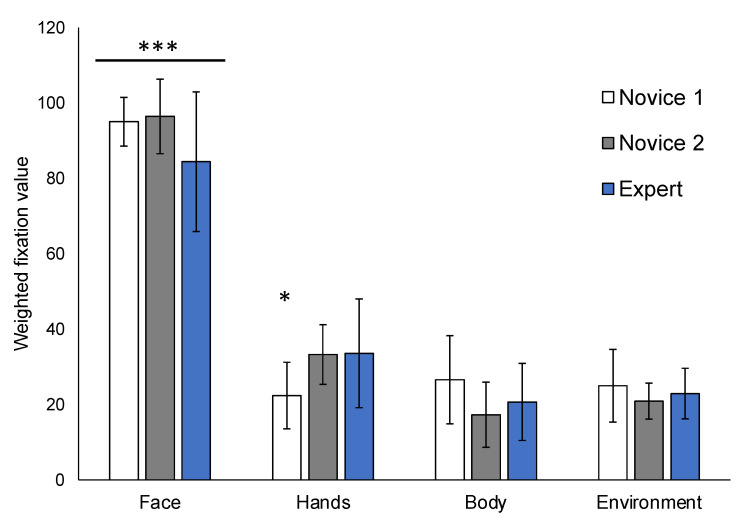
Average fixation order for target versus environment areas of interest among novice (Novice 1, Novice 2) and expert police. All participants fixated on faces earlier than hands, bodies, and the environment. Novice 1 participants fixated on hands significantly later than both Novice 2 and expert officers. *** *p* < 0.001, * *p* < 0.05.

Within-group analyses reveal that all participants fixated on suspect faces earlier than all other AOIs (hands: *Z* = −5.054, *p* < 0.001; body: *Z* = −5.089, *p* < 0.001; environment: *Z* = −5.088, *p* < 0.001), and that there were no significant differences between body and environment AOIs (*Z* = −0.966, *p* = 0.334). For Novice 1, there were no other differences in fixation order between hands, bodies, and the environment (*p* > 0.05). For Novice 2, hands were fixated earlier than bodies (*Z* = −3.181, *p* = 0.001) and the environment (*Z* = −2.668, *p* = 0.008). For Experts, hands were fixated earlier than the environment (*Z* = −2.295, *p* = 0.022) but not bodies (*Z* = −1.867, *p* = 0.062). The only significant between-group difference in fixation order was observed for the hands (*χ*^2^(2) = 8.001, *p* = 0.018), such that early novice participants recorded later fixations and received lower weighted values for this subAOI compared to both later novices (*U* = 22.5, *z* = −2.648, *p* = 0.008) and experts (*U* = 24.0, *z* = −2.188, *p* =0.029) (Figure 5).

## 4. Discussion

Utilizing objective gaze and fixation data, the current study measured early-stage SA (i.e., perception) among novice and expert police officers while they observed various images of staged police encounters. Overall, we found that all participants fixated longer on target persons compared to the surrounding environment, and that faces were fixated earlier than suspects’ hands or bodies. Furthermore, consistent with previous research [16], expert officers fixated earlier on hands compared to early and intermediate novice police trainees, and spent less time scanning the environment than early novices. By examining objective eye movement patterns while viewing typical police encounters, the current findings provide evidence-based insights for developing police training in SA and tactical decision-making. Training officers on optimal visual search strategies can enhance their SA and, in turn, minimize negative outcomes in operational field settings.

The finding that all participants fixated on targets longer and on their faces earliest compared to other areas of interest is perhaps unsurprising. People’s faces and facial expressions are highly salient features for human social interactions and, as demonstrated in our findings (Figure 5), for information gathering among police. Facial cues can provide information about an individual’s emotional and/or mental state, and the direction of a person’s gaze can signal their possible intentions and motives [25,26,27]. For example, a suspect constantly looking at their car’s glove compartment could signal a potentially hidden object, such as drugs or a weapon. Therefore, early fixation on target persons’ faces provides important information that can help inform officers’ predictions about what can happen next.

While the current findings suggest an optimal fixation order (target person’s face, hands, proximal environment), the ‘correct’ duration of fixation remains less prescriptive. That is, it may not always be better to spend less time scanning the environment, especially if the target person is actively moving and can access possible weapons from the environment. According to expert police instructors, optimal SA requires that officers control their gaze beyond automatic primitive responses and continue to evaluate (i.e., visually scan) the situation. In police tactics training, it is a well-known fact that the hands are the most dangerous physical feature of a target person. If a suspect wishes to seek harm against the police or another individual, they will most likely use their hands, either empty or with an object. By extension, a knife or firearm on the floor is not dangerous by itself; rather, these objects become immediately life-threatening if the officer does not see/perceive them (see below for further discussion on seeing versus perceiving) and the target person moves into the immediate vicinity of these instruments. For this reason, continuing to strategically scan the target person (including their face, hands, and waist) and their proximal environment is critically important for SA.

As demonstrated in the descriptive analysis (Figure 4), whether either or both groups attended more to the target or environment depends on the specific situational context of each picture. Importantly however, the gaze and fixation patterns between novice and expert police are consistent across all images, and also in line with previous findings [16]. Specifically, experts fixate significantly less on the environment and spend more time on the target (Figure 3). Considered together with the finding that early novices also fixate later on the hands (Figure 5), these untrained visuomotor patterns could potentially result in missing important cues related to the target person, further increasing risk of OSIs.

The current results show that after receiving at least 75% of basic tactical and use of force training (see [24]), the Novice 2 intermediate group fixated on hands earlier compared to Novice 1, and displayed gaze and fixation patterns that are more similar to experts (Figure 5). Therefore, progressing through the training program offered by the police agency [24], including reality-based use of force scenarios and training in basic tactics, has resulted in the development of more efficient visuomotor patterns among intermediate compared to early novices. In addition, eye tracking technology may be a suitable tool to track objective measures of learning and development for behaviors related to SA and tactical decision-making (see [13]).

### 4.1. Developing Expert Knowledge through Training

Through formal training and practical experience, experts develop improved behavioral strategies that become automatic and implicit. According to Klein’s Recognition Primed Decision Model [28], experts use their previous experiences to recognize patterns of cues (e.g., target person’s shifting gaze, nervous demeanor, putting hands in their waistband), and generate faster and more accurate predictions about possible next steps and outcomes. In the same way, visuomotor gaze patterns become refined as part of an experts’ motor repertoire, and elicit specific patterns of brain activity in regions responsible for object recognition [29] and motor planning [30]. In this way, visuomotor networks become more efficient following training [16]. By capitalizing on such learning-dependent plasticity, observation-based tasks can be an effective method for training (or re-training) early SA and tactical decision-making. Using a similar paradigm to the current study, officers can be presented with various staged images (or live-action videos) and receive direction on where to focus their attention based on tactically relevant information. Once basic visuomotor skills have been established in the learner’s repertoire, training can progress to more complex and stressful live scenarios [7,22,31].

To ensure that optimal SA strategies will be transferred to stressful field conditions, we recommend that SA training should also incorporate physiological stress management [12,31]. Police and other public safety personnel (PSP, e.g., firefighters, paramedics, corrections officers) experience significant physical and psychological disorders due to the highly stressful nature of their work [4,6,32]. Therefore, incorporating training that has multiple positive effects for performance and health would maximize limited resources while also achieving learning outcomes. Appropriate measures for evaluating performance and mental health in policing have been described elsewhere [13,33].

### 4.2. Seeing Is Not Perceiving: The Influence of Stress on Police Gaze, Fixation, and SA

Both applied and scientific research have shown that where an officer’s eyes and head are oriented, and what they consciously perceive or remember about a situation is impacted by stress. Under high threat training conditions, officers blink more, turn away, fixate less, and withdraw from target persons [3,17,20]. Following real-world critical incidents, officers report distorted sensory perceptions [18]. Several reviews also demonstrate how stress physiology exerts widespread effects on several cognitive and motoric processes, including memory [7] and motoric skills [34]. Accordingly, an officer’s eyes may fixate on an object but similar to the ’look but fail to see’ phenomenon identified in driving research [35], the officer may not consciously perceive it. At worst, an officer may misinterpret a benign object as a dangerous weapon [19], or vice versa (i.e., failing to perceive a real threat). According to SA theory and practice, information gathering does not end with visual perception: effective operators must understand how visual information can impact their next steps and inform decision-making (i.e., later stages of SA) [11].

Real-life police encounters are naturally different than still-image simulations. Given the stress-induced decrements to police gaze, fixation, SA, and performance outlined above, the current study was designed to investigate officers’ eye movement patterns during a non-stressful task. Experts were evaluated to derive tacit knowledge about implicit, automatic gaze behavior as a proxy for early-stage SA. Together with their qualitative responses to interview questions (see Methods), the current findings can provide a framework for expert SA in order to develop evidence-based SA training that directs novices’ attention and gaze to more tactically relevant areas.

### 4.3. Action versus Reaction: Time as a Critical Factor in Policing

In police work, officers must be prepared to make informed decisions in a matter of milliseconds, including the use of lethal force. These decisions can have significant consequences (i.e., life or death, physical or psychological injury), therefore, empirical investigation of the bases of officers’ decision-making (i.e., their perceptions) is crucial [7]. Especially in early SA, it is highly important to recognize what is immediately or potentially threatening to bystander(s), the target person, or the officer within the first 5 s of a situation. This includes identifying what is available to the target person by scanning the environment. It is interesting that such a small mean difference in visit duration in the environment (Novice 1 = 1.99 s, Novice 2 = 2.05 s, experts = 1.54, Figure 3) is statistically significant for the experts, but in a real-world context, many things can happen in 0.45 s, especially when it comes to high-stakes decision-making.

In a reaction time study by Blair et al. [36], it was found that officers were unable to shoot an armed suspect before they were shot at, even when officers were aiming at the target. According to fundamental time laws, reaction is always slower than action. Therefore, police officers should be trained to recognize and assess the situation as fast and as accurately as possible. When a situation is unfolding very quickly, officers must make decisions about their own behavior before things happen (that is, predicting what will happen before it even does) [1,11,28]. To achieve optimal early SA, evidence-based training of the visuomotor skills revealed in the current study can ensure that officers perceive the most critical information in an efficient and accurate manner. In turn, errors in judgement and decision-making that can lead to OSIs can be reduced.

### 4.4. Limitations

Out of 13 images presented in the current study, only a subset provided visit duration and fixation order data for two primary reasons. Firstly, due to technical limitations of the eye tracking apparatus, visit duration and fixation order data were not registered in small and/or adjacent AOIs (for example, see Figures S1 and S2 in the Supplementary Materials). Secondly, the images used in the current study were not designed for the specific purpose of an eye tracking study, but rather for the qualitative analyses that will be presented separately. For instance, the researchers were unaware that close proximity of hands, faces, and target items within a picture would interfere with the eye tracking methodology. Nonetheless, the current data generated results that were in line with other previous and very limited eye tracking studies in police [16]. Future research on scene perception among police should more deliberately consider placement and arrangement of targets as well as areas of interest to avoid such confounds.

As discussed above, eye tracking can only detect the physiological shift of gaze but not the shift of attention. Even though an individual’s eye may fixate on an object (e.g., bystander), they may not consciously perceive it [35]. Accordingly, missed visual cues will not be included in subsequent judgement, decision-making, or informed action. Therefore, future studies should also explicitly probe attention, understanding, and memory to understand their relationship to visual fixation.

Relative to eye tracking studies in non-specialist populations, the current study had small sample sizes, but is consistent with applied police research samples ([16]; for review see [7]). Additionally, this is an important preliminary dataset that compares an objective measure of early SA between expert officers and two levels of novice police trainees. Future research can continue to investigate the neurophysiological basis of police SA using carefully designed stimuli of static and/or moving images, simulating stressful operational conditions, and functional neuroimaging.

## 5. Conclusions

The purpose of the current study was to use experts’ automatic gaze behavior to identify what is ‘correct’ or ideal early-stage SA, as measured by fixation order and visit duration, while viewing typical police encounters. In line with previous eye tracking research in police, optimal SA involves less time scanning the environment (Figure 3), and more efficiently searching the target person’s hands beyond their facial expression (Figure 5). However, every situation is unique, and there is no single correct procedure or outcome. The goal of every encounter is to maintain personal and public safety by the best means possible.

The current study also highlights the importance of partnerships between police practitioners and scientists that can provide access to cutting-edge methodologies (i.e., eye tracking, neuroimaging) and guidance on designing effective experimental studies that answer targeted research questions that are directly relevant for police. Police and academic partnerships will also generate empirical knowledge that can be directly implemented into evidence-based training and practice that, in turn, minimize the occurrence of OSIs.

## Figures and Tables

**Table 1:** Demographic information.

Group.	*n* (Female)	Age *M* (*SD*)	Years of Experience *M* (*SD*)
Novice 1	10 (6)	25.6 (3.4)	<1.5 education
Novice 2	13 (5)	24.6 (4.4)	<1.5 education + tactical training
Experts	11 (0)	41.6 (4.3)	16.7 (3.9) duty, 8.0 (2.2) special units

## Data Availability

De-identified data supporting the reported results can be requested from the corresponding author.

## Supplementary Materials

The following are available online at https://www.mdpi.com/article/10.3390/ijerph19095047/s1, Figure S1: Picture 4 from experimental stimulus (excluded); Figure S2: Picture 7 from experimental stimulus (excluded); Figure S3: Picture 3 from experimental stimulus (target versus environment); Figure S4: Picture 8 from experimental stimulus (target versus environment); Figure S5: Picture 9 from experimental stimulus (target versus environment).
