# Supplementary material for: Experience-Dependent Effects to Situational Awareness in Police Officers: An Eye Tracking Study
Source: Int J Environ Res Public Health. 2022 Apr 21;19(9):5047. doi: 10.3390/ijerph19095047 (PMC9105864; doi:10.3390/ijerph19095047)
Supplement: Supplementary file 1 [file ijerph-19-05047-s001.zip › ijerph-1680661-supplementary.pdf]

Supplementary Materials

Experience-dependent changes to situational awareness in police: An eye tracking study

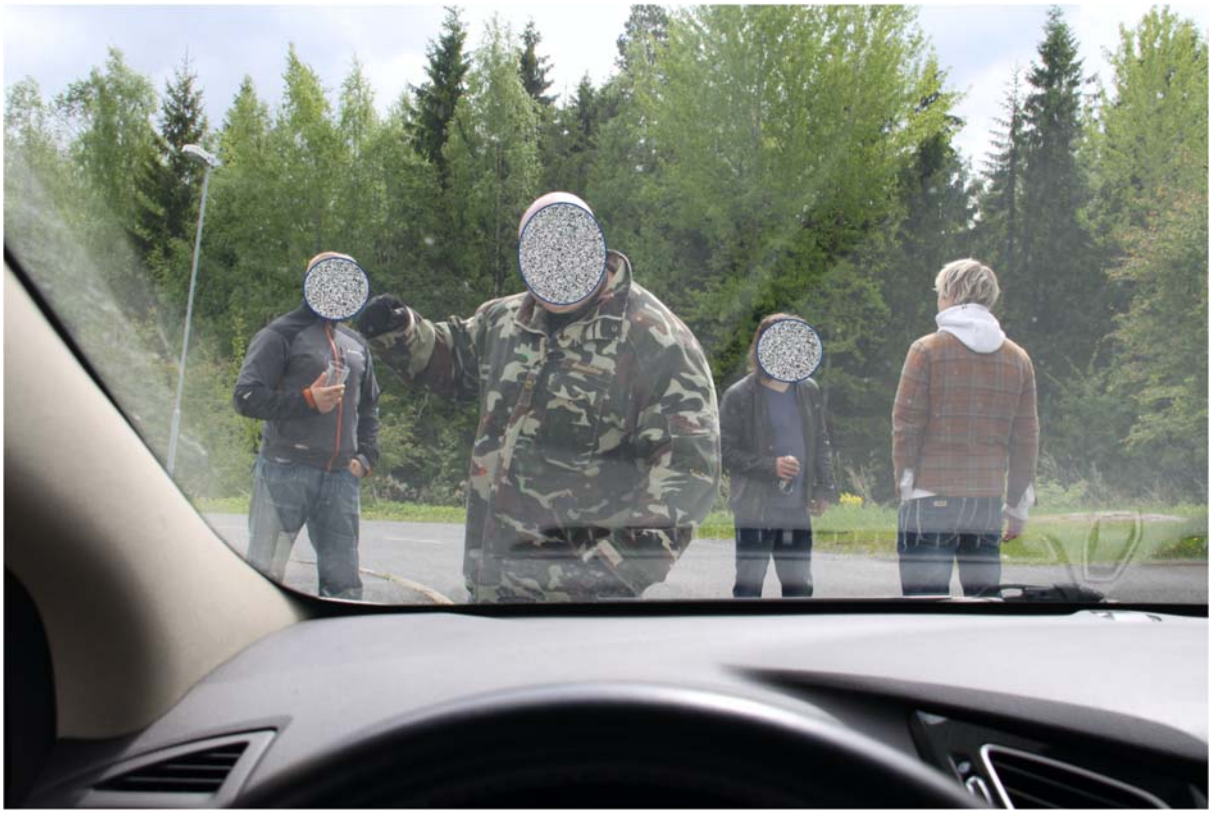

Figure S1: Picture 4 from experimental stimulus (excluded). This image was excluded from the visit duration and fixation order analyses due to significant overlap of areas of interest (AOIs) and a lack of sensitivity of the eye tracker system. For instance, the right hand of the suspect in the foreground overlapped too closely with the face of the suspect on the far left. Faces have been blurred to protect individuals' identity but were not blurred in the experiment.

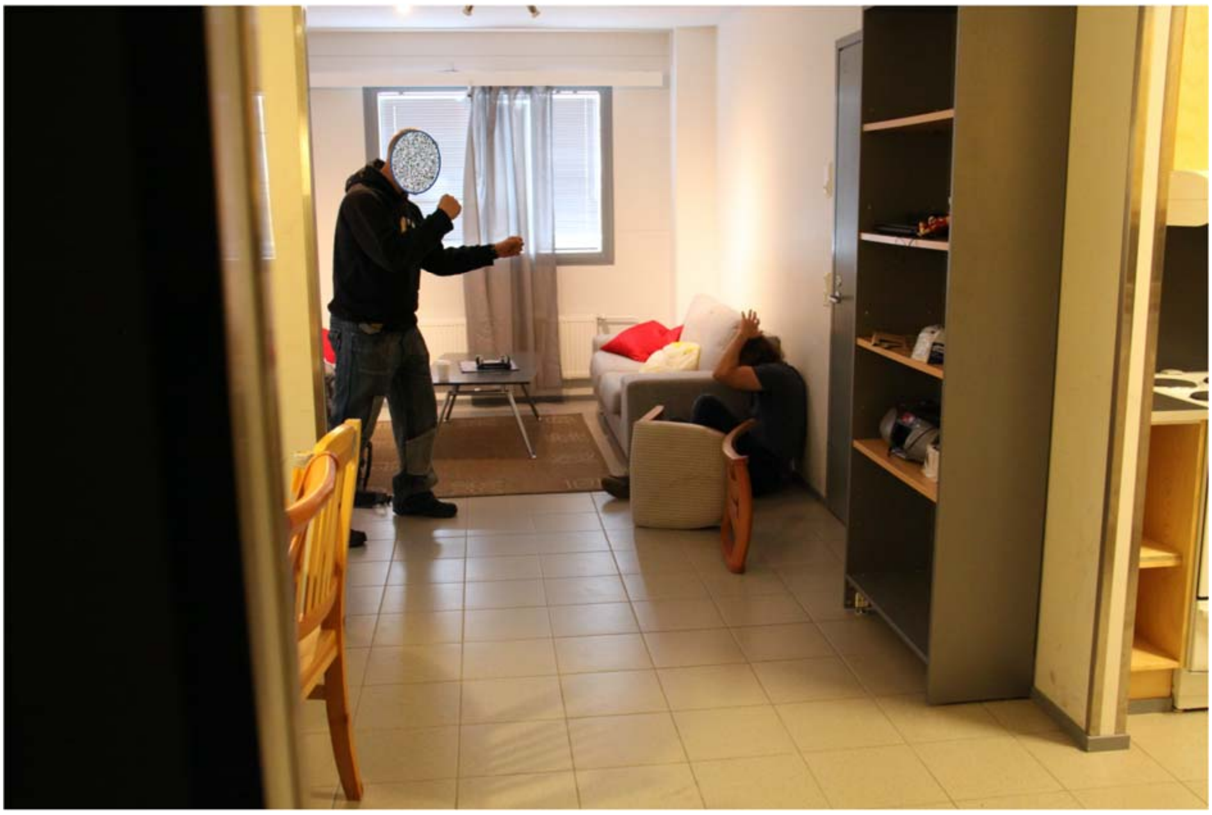

Figure S2: Picture 7 from experimental stimulus (excluded). This image was excluded from the fixation order analysis due to a lack of sensitivity of the eye tracker system to detect the sub-areas of interest (subAOIs) identified around the suspect person's (left) hands and face. However, the image was used in the overall target versus environment analysis of visit duration data. Faces have been blurred to protect individuals' identity but were not blurred in the experiment.

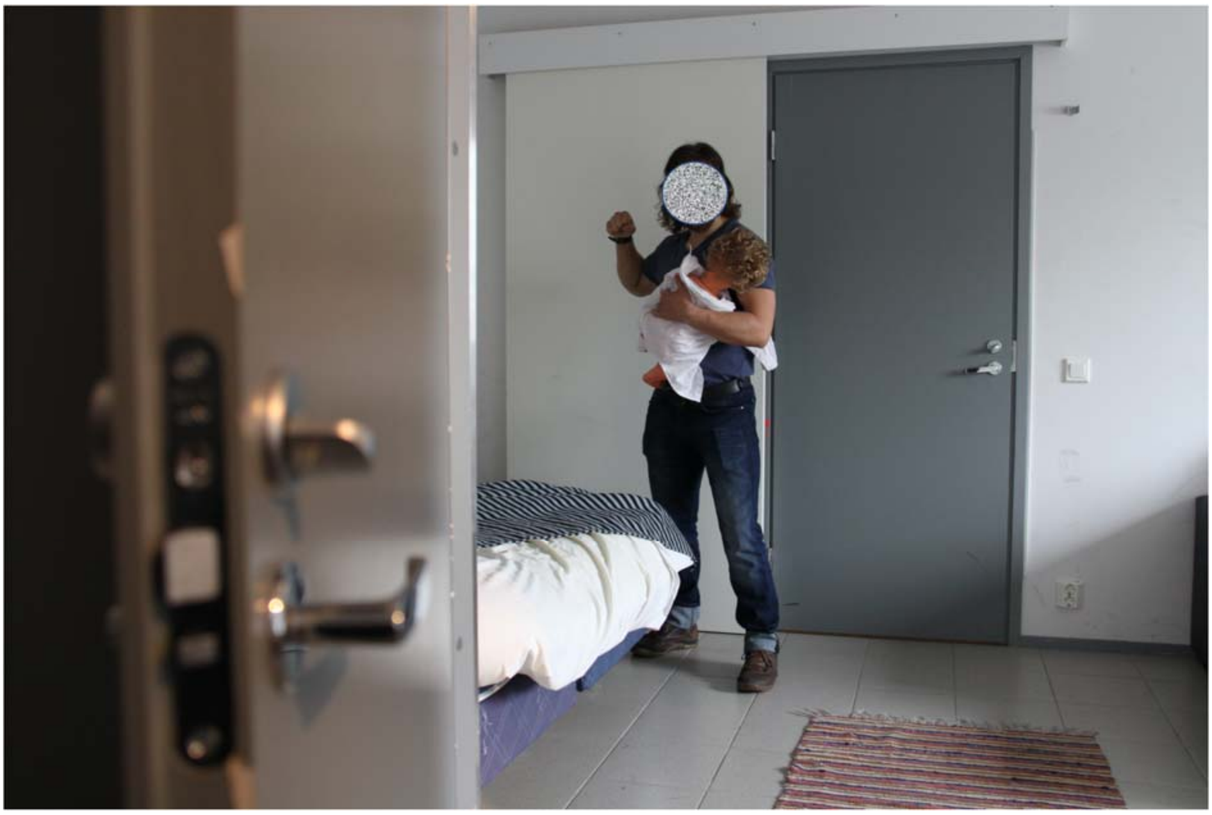

Figure S3: Picture 3 from experimental stimulus (target versus environment). All participants spent more time fixating on and scanning the area around the target person compared to the environment. Faces have been blurred to protect individuals’ identity but were not blurred in the experiment.

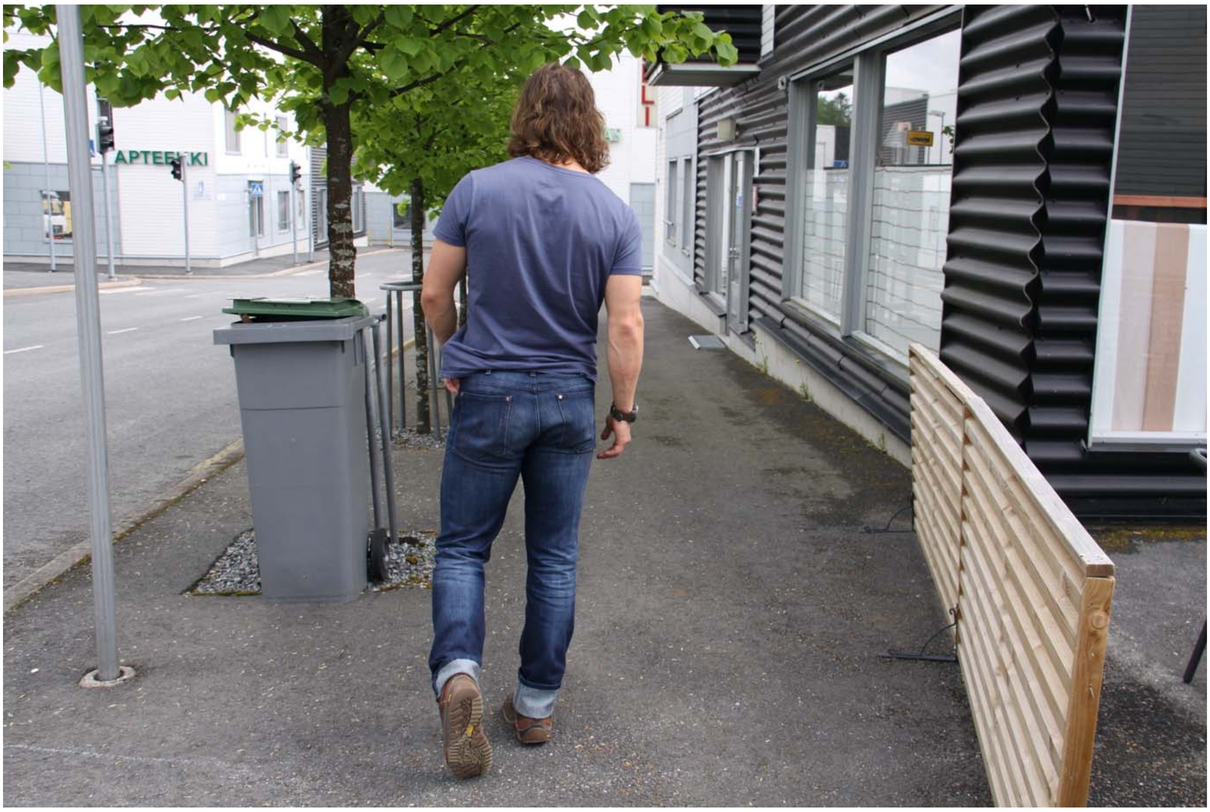

Figure S4: Picture 8 from experimental stimulus (target versus environment). All participants spent more time fixating on and scanning the area around the target person compared to the environment.

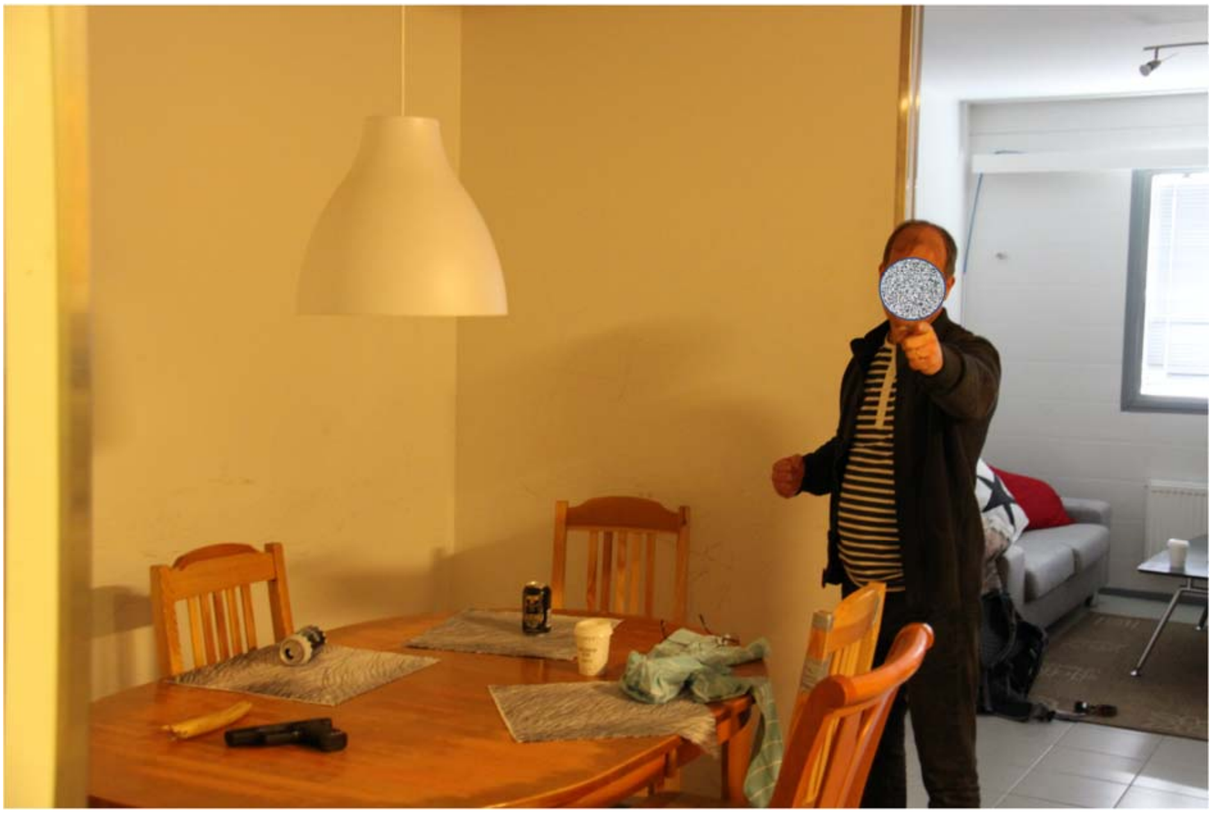

Figure S5: Picture 9 from experimental stimulus (target versus environment). All participants spent more time fixating on and scanning objects in the peripheral environment compared to the target person. Faces have been blurred to protect individuals' identity but were not blurred in the experiment.
